# Editorial: Methods in genitourinary oncology

**DOI:** 10.3389/fonc.2023.1274264

**Published:** 2023-08-24

**Authors:** Albert Jang, Avishay Sella, Amin H. Nassar, Vadim S. Koshkin

**Affiliations:** ^1^ Division of Solid Tumor Oncology, Department of Medicine, University Hospitals Seidman Cancer Center, Case Comprehensive Cancer Center, Case Western Reserve University, Cleveland, OH, United States; ^2^ Department of Oncology, Yitzhak Shamir Medical Center (Assaf Harofeh), Sackler School of Medicine, Tel-Aviv, Israel; ^3^ Department of Hematology/Oncology, Yale New Haven Hospital, New Haven, CT, United States; ^4^ Division of Hematology and Oncology, Department of Medicine, Hellen Diller Family Cancer Center, University of California San Francisco, San Francisco, CA, United States

**Keywords:** prostate cancer, bladder cancer, kidney cancer, research methods, geriatric oncology

The three most common genitourinary (GU) malignancies involving the bladder, kidney and prostate represent very distinct diseases, each with a unique pathophysiology and markedly different treatments. The diversity of these diseases is mirrored by the heterogeneity of the associated populations and treatments, and the variety of research methods used to approach them. Bladder, kidney and prostate cancers are all more common in the geriatric population, with many patients diagnosed in their seventh and eighth decades of life (1). Many geriatric patients have comorbidities that preclude the use of certain treatments and approved therapies, which in turn can compromise outcomes. Therefore, it is often critical to both identify patients who would benefit from more aggressive therapies, but also to account for their comorbidities. In this Research Topic of *Frontiers in Oncology* focusing on methods in GU oncology, we include five manuscripts describing varied research methods focusing on the importance of predicting patient outcomes prior to treatment initiation and incorporating personalized treatment options.

In the article by Singhal et al., the authors review the methods of the geriatric assessment (GA) for patients with GU cancers and provide suggestions for implementation in clinical GU studies. GA is a comprehensive assessment tool performed by a trained individual for adults 65 years and older to holistically evaluate their medical, psychosocial, and physical functioning prior to starting treatment (2). Prior studies have shown that patients with muscle-invasive bladder cancer (MIBC) undergoing radical cystectomy had a higher mortality risk if they had severe comorbidity as assessed by the Adult Comorbidity Evaluation 27 (ACE-27) (3) or if there was sarcopenia on CT scans. Nutritional status can be used as a surrogate for sarcopenia, based on the Malnutrition Universal Screening Tool (MUST) (4) or Patient-Generated Subjective Global Assessment (PG-SGA) (5). For patients with bladder cancer, cisplatin-based chemotherapy eligibility assessment must be performed using the Galsky criteria (6). In prostate cancer, the authors recommended the use of the Geriatric 8 (G8) questionnaire (7) to identify high-risk versus low-risk adults prior to treatment. For localized prostate cancer, the Charlson Comorbidity Index (8) should be followed prior to considering a radical prostatectomy. For metastatic prostate cancer, the Cancer and Aging Research Group Chemotherapy Toxicity Tool (CARG-TT) (9) can predict toxicity for chemotherapy and androgen deprivation therapy. For localized kidney cancer, on the other hand, the Charlson Comorbidity Index should also be used prior to considering a partial or radical nephrectomy. For metastatic kidney cancer, there was one pretreatment GA able to differentiate patients with greater toxicity risk with tyrosine kinase inhibitor therapy, but there were no definitive studies evaluating the reliability of GAs for predicting immunotherapy toxicity. Overall, the authors found published data regarding GA for patients with GU cancers to be limited, and significant challenges with implementing existing assessments due to lack of training and knowledge, lack of time, patient heterogeneity, and cumbersome assessment tools.

Three other articles described nomograms predicting outcomes for patients with kidney and bladder cancers. In the article by Liao et al., the authors queried the Surveillance Epidemiology and End Results (SEER) database for patients with clear cell RCC (ccRCC) from 2004-2015 (58,372 cases), in order to assess risk factors for ccRCC across seven different age groups. Patients were classified into seven age groups, and increasing age was associated with decreased cancer-specific survival (CSS) and overall survival (OS). Other independent risk factors included clinical, pathological, and social factors, such as grade, TNM (tumor, nodal, metastasis) stage, surgery, WHO/ISUP grade, gender, marital status, and race. Based on these factors, a nomogram was developed to predict CSS and OS. The authors stressed the importance of individualized treatment options for ccRCC based on all these risk factors and emphasized the need for a more detailed age grouping in studies. In the article by Li, S. et al., the authors similarly queried the SEER database for patients with chromophobe RCC (chRCC) from 2004-2015 (6,016 cases), and these cases were randomized 7:3 to training and validation cohorts, respectively. An external validation cohort of 249 patients with chRCC from 3 independent centers in Xuzhou, China was collected. chRCC is a rare non-clear cell variant of RCC with generally limited treatment options in the metastatic setting (10). Nomograms were created to predict post-operative CSS and OS, incorporating clinical, pathological and social factors as well. Factors associated with longer OS included younger age, female gender, being married, small tumor size, no radiation, and no chemotherapy. A factor associated with longer CSS included higher median household income. The authors concluded that the nomogram performed better than AJCC or TNM staging for predicting OS and CSS and could be successfully used to evaluate the prognosis of patients with chRCC. In the article by Li, L. et al., the authors developed and validated an MRI-based radiomics-clinical nomogram to individualize prediction for non-muscle-invasive bladder cancer (NMIBC) histologic grading (low- versus high-grade). This analysis included 169 consecutive patients with pathologically confirmed NMIBC who underwent MRI scans from September 2017 through December 2021, and were randomized in a 7:3 ratio to training and validation cohorts. The gold standard for assessing NMIBC grade is with cystoscopy and biopsy, but there are significant limitations to this approach, which is invasive, expensive, and may not capture the entire tumor. Using MRI radiomics features and clinical factors (age, sex, tumor size, number of tumors), a radiomics-clinical nomogram was developed to predict the probability of NMIBC grade. The nomogram was successfully used to predict high-grade NMIBC, which urologists can utilize in deciding whether to conduct further invasive procedures.

The final article of this Research Topic involved treatment. Bi et al. analyzed the safety and efficacy of iodine-125 intraluminal brachytherapy as localized and palliative treatment for ureteral carcinoma in 22 patients who were not suitable for either surgical resection or systemic therapy. Included patients were treated between November 2014 and November 2021, with 46 total seed strand sessions. Surgical resection is the usual standard of care for this disease, and external radiotherapy can also be used (11). However, both have associated risks and adverse events which many patients may not tolerate. In this analysis, iodine-125 intraluminal brachytherapy resulted in a 64% disease control rate at 6-months, median progression-free survival of 13.0 months and median OS of 24.7 months. The authors concluded that iodine-125 seed strand therapy may be an alternative treatment option for patients who are not candidates for surgical resection or systemic therapy.

Manuscripts included in this Research Topic highlight the diversity of research methods in GU oncology which mirror the dynamic treatment landscape in this disease space over the past 20 years. Research in GU oncology is increasingly focusing on individualizing patient treatment options and identifying patients most likely to respond to specific therapies. Future directions include identifying and implementing research methods better suited to moving this field forward, as we continue to advance the care of patients with GU malignancies.

## Author contributions

AJ: Conceptualization, Data curation, Methodology, Project administration, Writing – original draft, Writing – review & editing. AN: Conceptualization, Data curation, Supervision, Writing – review & editing. AS: Data curation, Supervision, Writing – review & editing. VK: Conceptualization, Data curation, Methodology, Project administration, Supervision, Writing – original draft, Writing – review & editing.
